# A high proportion of red snapper sold in North Carolina is mislabeled

**DOI:** 10.7717/peerj.9218

**Published:** 2020-06-25

**Authors:** Erin T. Spencer, Emilie Richards, Blaire Steinwand, Juliette Clemons, Jessica Dahringer, Priya Desai, Morgan Fisher, Sloane Fussell, Olivia Gorman, Diamond Jones, Amanda Le, Kayla Long, Cammie McMahan, Caitlin Moscarito, Catherine Pelay, Erica Price, Anna Smith, Allison VanSant, John F. Bruno

**Affiliations:** 1 Environment, Ecology, and Energy Program, University of North Carolina at Chapel Hill, Chapel Hill, NC, United States of America; 2 Department of Biology, University of North Carolina at Chapel Hill, Chapel Hill, NC, United States of America

**Keywords:** Red snapper, Fisheries, Seafood mislabeling, DNA barcoding

## Abstract

Seafood mislabeling occurs when a market label is inaccurate, primarily in terms of species identity, but also regarding weight, geographic origin, or other characteristics. This widespread problem allows cheaper or illegally-caught species to be marketed as species desirable to consumers. Previous studies have identified red snapper (*Lutjanus campechanus*) as one of the most frequently mislabeled seafood species in the United States. To quantify how common mislabeling of red snapper is across North Carolina, the Seafood Forensics class at the University of North Carolina at Chapel Hill used DNA barcoding to analyze samples sold as “red snapper” from restaurants, seafood markets, and grocery stores purchased in ten counties. Of 43 samples successfully sequenced and identified, 90.7% were mislabeled. Only one grocery store chain (of four chains tested) accurately labeled red snapper. The mislabeling rate for restaurants and seafood markets was 100%. Vermilion snapper (*Rhomboplites aurorubens*) and tilapia (*Oreochromis aureus* and *O. niloticus*) were the species most frequently substituted for red snapper (13 of 39 mislabeled samples for both taxa, or 26 of 39 mislabeled total). This study builds on previous mislabeling research by collecting samples of a specific species in a confined geographic region, allowing local vendors and policy makers to better understand the scope of red snapper mislabeling in North Carolina. This methodology is also a model for other academic institutions to engage undergraduate researchers in mislabeling data collection, sample processing, and analysis.

## Introduction

Seafood mislabeling is a widespread problem and can occur at any step in the seafood supply chain. Unintentional mislabeling can result from loss of information in the supply chain or misinformation, such as assigning closely related species to a singular common vernacular name (Warner, Timme & Lowell, 2012). Intentional mislabeling can be used to hide the identity of illegally-caught species or increase the price of less-desirable or cheaper species, such as substituting farmed tilapia for wild-caught red snapper (*Lutjanus campechanus)* (Spencer & Bruno, 2019).

Species substitution could lead to overexploitation of at-risk fish populations, especially when used as a method to mask the sale of illegally-caught species. Following a nationwide ban in Belize on harvesting herbivorous fish, a 2012 study found 32–51% of fillets in seafood markets were mislabeled, and 5–7% were illegally-caught herbivorous species like parrotfish (Cox et al., 2012). Another study of almost 200 “lemon shark” fillets in New Zealand found 40% were illegally-harvested species such as hammerheads, school sharks, and bronze whalers (Smith & Benson, 2001). Another recent study of fish and chips vendors in the United Kingdom found the majority of samples were actually spiny dogfish (*Squalus acanthias*), a species of shark whose population is endangered in the Northeast Atlantic (Hobbs et al., 2019). Additionally, if a species appears to be readily available in the market, it creates the perception of an abundant stock, regardless of the true stock status (Marko et al., 2004). This is particularly important for species like red snapper, whose stock is still below target population levels (SEDAR, 2016). If a consumer continues to buy a mislabeled species in the market, this could increase the demand for that species overall, which in turn could incentivize illegal harvest and continued mislabeling (Cox et al., 2012).

There are multiple instances of mislabeled seafood threatening human health. For example, two people in Chicago became sick after eating poisonous pufferfish mislabeled as “monkfish”, and over 600 people in Hong Kong became ill after eating “cod” that was really escolar, a fish that in large quantities can cause severe diarrhea (Jacquet & Pauly, 2008). Fraud also compromises a consumer’s ability to adhere to dietary restrictions, such as avoidance by pregnant women of high-trophic level species that could contain elevated mercury levels (Marko, Nance & Van den Hurk, 2014; Rasmussen, Nettleton & Morrissey, 2005).

Red snapper is one of the most commonly mislabeled species in the United States (Table 1) (Marko et al., 2004; Willette et al., 2017). Only *Lutjanus campechanus* is allowed to be labeled as red snapper under Federal Drug Administration (FDA) guidelines (Warner, Timme & Lowell, 2012). A Los Angeles study found a 100% mislabeling rate of red snapper in restaurants, where it was commonly replaced with red seabream, yellowtail snapper, and amberjack (Willette et al., 2017). Another study across eight states found 17 of 22 samples mislabeled (77%), commonly replaced by lane or vermilion snapper. Although red snapper is native to North America, half of the samples were from other regions of the world (Marko et al., 2004). A recent study of regional red snapper mislabeling in the Southeastern United States found similar results; an overall mislabeling rate of 72.6%, with samples from North Carolina mislabeled 90% of the time (Spencer & Bruno, 2019).

**Table 1:** Summary of reported red snapper mislabeling rates from the United States.

**Study**	**Location sampled**	**Total number of samples**	**Percent mislabeled**
Marko et al. (2004)	Vendors in Delaware, Florida, Illinois, Massachusetts, New York, North Carolina, South Carolina, Wisconsin.	22	77.3
Warner et al. (2013)	Restaurants, sushi venues, grocery stores and seafood markets across the United States.	120	94.2
Khaksar et al. (2015)	Wholesalers, retailers, and resturants in New York, Texas, California.	16	100.0
Willette et al. (2017)	Sushi restaurants and high end grocery stores in Los Angeles, California.	32	100.0
Spencer & Bruno (2019)	Sushi restaurants, grocery stores, and seafood markets in North Carolina, South Carolina, Georgia, and Florida.	62	72.6
Current study	Sushi restaurants, grocery stores, and seafood markets in North Carolina.	43	90.7

The seafood industry has major cultural and economic significance in the state of North Carolina. In 2015, commercial fishermen sold 66 million pounds of finfish and shellfish, resulting in a dockside value of $104 million (Smith, 2016). Seafood imports to North Carolina have increased almost 70% from 1996 to 2007, and competition from imported seafood is considered one of the top threats to North Carolinian fishers (Newsome, 2014). To combat this, there is a growing movement in North Carolina to “eat local fish”, with the goal of reversing the decline of the seafood industry. A regional survey found consumers buy North Carolina seafood in part because they value the “heritage of fishing communities” and “the lifestyle of fishermen” (Nash, 2015). Although red snapper is a wild-caught species native to North Carolina, many “red snapper” samples collected in North Carolina in a recent study were species that are either imported or farmed, and all mislabeled samples were less expensive species (Spencer & Bruno, 2019).

Previous studies have either assessed mislabeling over a very broad or very narrow geographic region, such as an entire country or single city. As mislabeling rates can vary by species and location (Warner et al., 2013), thorough sampling of a specific species from multiple vendors at a regional level gives a more defined view of labeling patterns of that particular seafood product. Understanding the scope and frequency of red snapper mislabeling in North Carolina will help local officials and consumers pinpoint sources of mislabeling, and thus develop regionally-specific policies to combat the problem.

The purpose of this study was to quantify the rate of red snapper mislabeling across North Carolina and to model how to engage students in the sample collection and analysis process. The Seafood Forensics class at the University of North Carolina at Chapel Hill used standard DNA barcoding methods to determine the true species identity of “red snapper” samples purchased from sushi restaurants, seafood markets, and grocery stores in ten counties within North Carolina. We defined mislabeling as when a fish was sold under an incorrect species name. We hypothesized there would be a significant difference between mislabeling rates at each of the three vendor types, in concordance with other red snapper mislabeling studies in different geographic regions.

## Methods

### Sample collection

UNC-Chapel Hill’s Seafood Forensics class is a course-based undergraduate research experience (CURE) that aims to expose students to research while providing opportunities to collaborate, problem solve, and make discoveries (Gin et al., 2018). The 16 students enrolled in this course met for four hours once a week over the semester and collected 52 samples between August 2017 and November 2017. Only one sample was collected from each vendor, and samples were a mix of fillets, whole fish, and nigiri or sashimi labeled as “red snapper”. The class collected 19 samples from 19 different sushi restaurants, 20 samples from four grocery store chains and four independent grocery stores (20 different physical locations), and 13 samples from 13 different seafood markets located in Cumberland, Durham, Forsyth, Guilford, Mecklenburg, Orange, Pitt, Wake, Watauga, and Wilson counties. As sampling took place as part of the class, sampling locations were selected based on proximity to Chapel Hill, North Carolina, and availability of red snapper products. Any samples physically labeled as “red snapper” were collected, whereas samples labeled “snapper” had to be verbally confirmed by a vendor employee to be red snapper prior to collection. Samples were stored in Ziploc bags and frozen until extraction.

### DNA extraction protocol

We used a DNA barcoding protocol modified from Willette et al. (2017). DNA barcoding is a widely-used method to determine seafood mislabeling, as it amplifies the mitochondrial cytochrome *c* oxidase 1 (CO1) gene which is well-conserved among fish species, allowing for identification to the species level (with the exception of some closely-related species) (Khaksar et al., 2015). We extracted genomic DNA from 20 mg of thawed fish tissue using the Qiagen DNeasy Blood and Tissue Kit Protocol (Qiagen, Inc.). The samples were incubated in 20 µL Proteinase K digest for 1–24 h and the DNA was eluted in 20 µL distilled water. A ∼650 bp fragment of the CO1 gene was amplified following cycling protocol from Willette et al. (2017), with the exception of initial denaturation at 95 °C instead of 94 °C, as outlined by Willette et al. The primer cocktail used was designed in Ivanova et al. (2007) (C_FishF1t1 and C_FishR1t1). PCR amplification was done with 1 µl of the extracted DNA, 1.5 µl of 10 MM of each of the four primers, and 17 µl of molecular grade water in Illustra Pure Taq Ready-To-Go PCR bead tubes. A tube with 1 extra µl of water and no added DNA was also prepared as a control to ensure none of the DNA had contaminated the PCR reagents. The thermal cycling (conducted in Bio-Rad T100 Thermal Cycler) began with an initial denaturation phase of 95 °C for 5 min, followed by 35 cycles of denaturation at 94 °C for 30 s, annealing at 50 °C for 45 s, and extension at 72 °C for 60 s. All of this was followed by a final extension phase at 72 °C for 10 min. PCR amplification success was confirmed by agarose gel electrophoresis and successful PCR products were sent to the Eton BioScience facility (Raleigh, NC) for purification and Sanger sequencing, using the M13F primer from Ivanova et al. (2007) as the sequencing primer.

### Analysis

We used 4Peaks software to visualize chromatograms of the sequenced gene region for each sample and selected a length of clean sequence with clear base pair calls (typically around 450 bp) and compared this to CO1 sequences from known specimens on two platforms: (1) the National Center for Biotechnology Information (NCBI) Genbank database using the Basic Local Alignment Search Tool (BLAST) on the NCBI website (http://blast.ncbi.nlm.nih.gov/Blast.cgi), and (2) the Barcode of Life Data Systems (BOLD) Identification engine (http://www.boldsystems.org/index.php/IDS_OpenIdEngine). Our samples were then identified to the lowest taxonomic level possible using a 98% query cover and percent identity match for nucleotide homology with known sequences on the database. Samples that were identified under the FDA approved scientific name for red snapper, *Lutjanus campechanus,* or *L. purpureus* were considered correctly labeled. We also considered the one sample identified as southern red snapper (*Lutjanus purpureus*) to be correctly labeled given convincing evidence that *L. campechanus* and *L. purpureus* comprise a single species of red snapper in the western Atlantic (Gomes, Sampaio & Schneider, 2012). All other species matches were determined to be mislabeled samples. We used a chi-square test with an alpha of 0.05 in R studio to determine if there was a significant difference between frequency of mislabeling by vendor type. Study data are publicly available here: https://doi.org/10.6084/m9.figshare.11971461.

## Results

Positive species identification was obtained for 43 of the 52 samples (all identifications were based on top matches with ≥98% sequence similarity). Of the 43 identified samples, 14 were from grocery stores, 12 were from smaller seafood markets, and 17 were from sushi restaurants. In total, 90.7% (39 of 43 samples) were mislabeled. Almost a quarter of substituted species (10 of 43 samples) were of the same genus as red snapper (*Lutjanus*) and 58% (25 of 43) were of the same family (Lutjanidae) (Table 2). All sushi restaurant samples were mislabeled, with 76.5% (13 of 17 samples) identified as Nile or blue tilapia. All seafood market samples were mislabeled (*n* = 12) and all were identified as vermilion snapper. Of grocery store samples, 71.4% (10 of 14 samples) were mislabeled, and substituted species included vermilion, lane or spotted rose snapper, yellowtail snapper, and crimson or saddletail snapper. There was a statistically significant difference in mislabeling rates between vendor types (Chi square, *p* = 0.010, *α* = .05).

## Discussion

Our results indicate that “red snapper” purchased from ten counties within North Carolina from a variety of vendor types is rarely *Lutjanus campechanus*: 90.7% of all the samples (*n* = 43) were mislabeled, and in seafood markets and sushi restaurants the mislabeling rate was 100%. These values are similar to frequencies of red snapper mislabeling reported by other studies (Table 1). All of the correctly labeled red snapper samples came from a single national grocery store chain that emphasizes seafood traceability in their marketing. All four locations for that chain that we sampled across North Carolina correctly labeled their red snapper fillets.

Sushi restaurants, for which the mislabeling rate was 100%, were the only vendor type in which tilapia was sold as a substitute for red snapper. All 12 samples purchased from seafood markets were vermilion snapper, a fish that has similar body shape and color to red snapper. Vermilion snapper are closer to their target population level in the South Atlantic and reach sexual maturity at a younger age than red snapper, and are less sensitive to fishing pressure (SEDAR, 2012). They are, however, considered vulnerable by the International Union for Conservation of Nature (IUCN) and populations could decline if fishing pressure increases (IUCN, 2018). If mislabeling of these samples occurred before the fish were landed and counted on the dock, landing data could be artificially low for substituted species like vermilion snapper (Di Pinto et al., 2015). This could potentially allow overharvest of substituted species to go unrecognized and unregulated (Carvalho et al., 2011; Cawthorn, Baillie & Mariani, 2018).

**Table 2:** The species identities of 43 processed samples. For some recent sister species, even rapidly evolving regions like the CO1 gene do not have enough genetic differentiation to be able to easily distinguish between species. In these cases, samples were noted as being either species (e.g., Northern and southern red snapper (*Lutjanus purpureus/campechanus*) and rose and lane snapper (*Lutjanus guttatus/synagris*).

**Name**	**Scientific name**	**Count**
Vermilion snapper	*Rhomboplites aurorubens*	13
Nile tilapia	*Oreochromis niloticus*	12
Rose/lane snapper	*Lutjanus guttatus/synagris*	8
Red snapper	*Lutjanus purpureus/campechanus*	4
Malabar blood/crimson snapper	*Lutjanus malabaricus/erythropterus*	2
Australasian snapper/red seabream	*Pagrus auratus/major*	2
Blue tilapia	*Oreochromis aureus*	1
Yellowtail snapper	*Ocyurus chrysurus*	1

Although these results provide valuable information regarding the scope of mislabeling in North Carolina, sampling the vendors alone does not tell the whole story. Seafood mislabeling can occur anywhere along the supply chain, including by the distributor Shehata et al., 2019. Information about each sampled vendor’s supplier could help understanding of where along the supply chain mislabeling is occurring. It could also help to clarify a potential bias in our study—a geographically smaller sample region could mean that a few distributors are supplying fish to some or all of our vendors. A high rate of mislabeling at the distributor level in North Carolina could result in mislabeling rates that are higher across vendors than other areas of the same size but that have a different, or more diverse, set of distributors.

This study, as well as a number of other recent mislabeling studies (Warner, Timme & Lowell, 2012; Galal-Khallaf et al., 2014; Spencer & Bruno, 2019) collected samples over only a few months. Future studies should evaluate the relationship between fishery season and mislabeling rates. During the sample period, the red snapper commercial fishery was not in season in the South Atlantic. The last day of sampling was the opening day of the commercial season in North Carolina. Determining if there is a difference in mislabeling rate depending on whether the fish is in season or not could help target enforcement during times of the year when mislabeling rates are highest.

## Conclusion

We found widespread mislabeling of red snapper samples collected in North Carolina, especially in sushi restaurants and seafood markets. This study adds to a growing body of research that suggests high levels of seafood mislabeling, especially of red snapper. Studies to date span geographic regions and indicate that despite increased awareness about the prevalence of seafood mislabeling, little has been done to effectively curb rates of mislabeling. Marko et al. first brought international attention to the issue in 2004, and if anything, mislabeling of this species has increased. Continued research into the frequency and scope of mislabeling on local, regional, and national scales, and across seafood types, will give scientists and policy makers a better picture of those markets most at-risk for mislabeling. However, until stronger action is taken to monitor seafood and enforce labeling regulations, it is likely that rates will continue to remain high, putting both human and environmental health at risk.

##  Supplemental Information

10.7717/peerj.9218/supp-1 Supplemental Information 1 List of seafood sample true identities

10.7717/peerj.9218/supp-2 Supplemental Information 2 UNC-CH seafood forensics class red snapper study data

10.7717/peerj.9218/supp-3 Supplemental Information 3 GenBank sequences

## Additional Information and Declarations

### DNA Deposition

The following information was supplied regarding the deposition of DNA sequences: The sequences and GenBank accession numbers are accessible as a Supplemental File and at Bruno, John; Spencer, Erin (2020): UNC-CH seafood forensics class red snapper study data. figshare. Dataset. 10.6084/m9.figshare.11971461.v5.

### Data Availability

The following information was supplied regarding data availability: The data available in the Supplementary Files shows the vendor type, whether or not the sample was mislabeled, and the true identity of the sample.
